# Omic characterizing and targeting gut dysbiosis in children with autism spectrum disorder: symptom alleviation through combined probiotic and medium-carbohydrate diet intervention - a pilot study

**DOI:** 10.1080/19490976.2024.2434675

**Published:** 2024-12-04

**Authors:** Yalin Li, Weiwei Hu, Bing Lin, Teng Ma, Zhentian Zhang, Weiqian Hu, Rui Zhou, Lai-Yu Kwok, Zhihong Sun, Cuifeng Zhu, Heping Zhang

**Affiliations:** a Inner Mongolia Key Laboratory of Dairy Biotechnology and Engineering, Inner Mongolia Agricultural University, Hohhot, Inner Mongolia, China; b Key Laboratory of Dairy Products Processing, Ministry of Agriculture and Rural Affairs, Inner Mongolia Agricultural University, Hohhot, Inner Mongolia, China; c Key Laboratory of Dairy Biotechnology and Engineering, Ministry of Education, Inner Mongolia Agricultural University, Hohhot, Inner Mongolia, China; d Department of Clinical Nutrition Shenzhen Hospital, Southern Medical University, Guangdong, China; e Shenzhen Key Laboratory of Gastrointestinal Microbiota and Disease, Shenzhen Hospital of Southern Medical University, Shenzhen, Guangdong, China

**Keywords:** Gut microbiota, phage, virome, fecal metabolome, childhood autism rating scale, gastrointestinal symptom rating scale

## Abstract

Autism spectrum disorder (ASD) currently lacks effective diagnostic and therapeutic approaches. Disruptions in the gut ecosystem have been observed in individuals with ASD, suggesting that targeting gut microbiota through probiotic and dietary supplementation may serve as a potential treatment strategy. This two-phase study aimed to characterize the fecal metagenome of children with ASD and investigate the beneficial effects of a combined probiotic and medium-carbohydrate intervention in ASD. Fecal metagenomes of children with ASD were compared to those of typically developing children, revealing intestinal dysbiosis in ASD, characterized by reduced levels of *Prevotella* sp. *Dialister invisus*, and *Bacteroides* sp. along with increased predicted abundances of inosine, glutamate, xanthine, and methylxanthine. The gut bacteriome and phageome exhibited high cooperativity. In a 3-month pilot study, *Bifidobacterium animalis* subsp. *lactis* Probio-M8 (Probio-M8) was administered alongside a medium-carbohydrate diet to Chinese children with ASD. The primary endpoint was the Childhood Autism Rating Scale (CARS), while the secondary endpoint was the Gastrointestinal Symptom Rating Scale (GSRS). A total of 72 autistic children were initially recruited for the intervention study, but only 53 completed the intervention. Probio-M8, in combination with dietary intervention, significantly improved CARS and GSRS scores, increased fecal levels of *Bifidobacterium animalis*, *Akkermansia muciniphila*, *Fusicatenibacter saccharivorans*, and *Sutterella* sp. while also reducing *Blautia obeum* (Benjamini-Hochberg corrected *p* ≤ 0.05 for all cases). The intervention also modulated fecal metabolites associated with the metabolism of amino acids (lysine), neurotransmitters (glutamate, γ-aminobutyric acid), polyunsaturated fatty acids (arachidonate, myristic acid), and vitamin B_3_. In conclusion, Probio-M8 combined with medium-carbohydrate diet effectively improved ASD symptoms, with associated changes in the gut microbiome and metabolome, supporting its potential as an adjunctive therapy for ASD.

## 1. Introduction

Autism spectrum disorder (ASD) is a complex neurodevelopmental condition, characterized by impaired social interaction, communication difficulties, and restricted behaviors. Its etiology is multifaceted, involving immune dysregulation, genetic predisposition, and environmental influences.^1^ The global prevalence of ASD is 1–2%, with a male-to-female ratio of 5:1.^2,3^ In China, an estimated 3 to 5 million children are affected.^4^ The complex pathogenesis and individual heterogeneity of ASD often result in missed opportunities for early intervention, exacerbating the condition and impacting the quality of life for individuals and their families. Gastrointestinal symptoms such as abdominal pain, constipation, and diarrhea are prevalent in 55% of children with ASD and correlate positively with ASD severity.^5^ Moreover, gut microbiota transfer from individuals with ASD to mice can induce autism-like behavior in the recipients, suggesting a potential role for gut microbiota in ASD pathogenesis.^6^ The rising prevalence of ASD and its complex etiology have sparked extensive research efforts to identify effective interventions.

The human gastrointestinal tract hosts over 10^14^ microorganisms, forming the gut microbiota, which is vital for immune function and intestinal health. For example, previous studies have shown increased Firmicutes and decreased Bacteroidetes in mice with autism-like behaviors^7^ as well as significant differences in gut microbiota between ASD patients and healthy individuals, including higher Firmicutes/Bacteroidetes ratios and reduced abundance of beneficial bacteria, such as *Bifidobacterium* and *Blautia* .^8,9^ Positive correlations exist between certain microbes, such as *Enterobacter* and *Shigella*, and gastrointestinal symptoms in ASD. Fecal transplants have been effective in restoring gut microbiota diversity and reducing autism symptoms.^10,11^ The gut microbiota interacts with the brain via the gut-brain axis, and disruptions in this communication may lead to neurological disorders.^12^ For instance, certain gut microbe-originated metabolites like intestinal 5-hydroxytryptamine (5-HT), taurine, and 4-ethylphenol sulfate can impact the brain through the enteric nervous system and vagus nerve pathways. Patients with ASD often have lower levels of short-chain fatty acids (SCFAs), except for propionic and acetic acids. Notably, propionic acid can cross the blood-brain barrier and influence serotonin levels, potentially affecting autistic behaviors.^13,14^

Around 90% of children with autism exhibit problematic eating behaviors, such as food allergies and picky eating, which can reduce dietary and microbiota diversity.^15^ Restrictive diets, such as ketogenic, gluten-free, and casein-free diets, have been used to treat neurodevelopmental disorders including autism, but may not be difficult to implement due to children’s resistance to dietary changes. Given the limitations of these diets, microbial therapy using probiotics has emerged as a promising strategy to restore gut microbiota and improve neurological conditions.^16,17^ Probiotics, especially psychobiotics, can enhance mental health benefits by interacting with gut bacteria to influence neurotransmitter secretion and modulate the gut-brain axis.^18^ For instance, specific probiotics like *Bifidobacterium longum* CCFM1077 have been shown to alleviate autistic behaviors in mice by regulating neurotransmitters and mitigating microglia activity in the cerebellum.^19^ Positive results have also been observed in autistic patients from a double-blind, placebo-controlled trial, where treatment with multiple probiotic strains for six months improved gastrointestinal symptoms, adaptive functioning, and sensory profiles.^20^ Supplementation with *Lactobacillus acidophilus*, *Lacticaseibacillus rhamnosus*, and *Bifidobacterium longum* increased beneficial gut bacteria, leading to significant reductions in autism severity and gastrointestinal issues.^21^ While some studies have not found significant benefits, the potential for probiotics to effectively and safely improve ASD symptoms remains promising.

This study was conducted in two phases: the first aimed to characterize the ASD fecal metagenome, while the second was an open-label, single-arm pilot study examining the effects of *Bifidobacterium animalis* subsp. *lactis* Probio-M8 (Probio-M8) combined with a balanced diet in Chinese children with ASD. Probio-M8, known for its gastrointestinal tolerance^22^ and efficacy in treating autism in mice,^23^ was used alongside a structured medium-carbohydrate meal plan. The meal plan aimed to standardize dietary intake and provide balanced nutrition for children with ASD during the intervention. We monitored changes in the Childhood Autism Rating Scale (CARS) and the Gastrointestinal Symptom Rating Scale (GSRS), as well as alterations in the gut microbiome and metabolome, both before and after the intervention. The findings of this study will enhance our understanding of probiotic and diet interventions for ASD and aid in developing new treatments.

## 2. Materials and methods

### 2.1. Trial design and subject recruitment

This prospective study employed a single-arm, open-label design over three months. Initially, 85 patients diagnosed with ASD were recruited. Following a two-week screening period, some were excluded for reasons such as not meeting inclusion criteria, declining to participate, or other factors (Figure S1). Ultimately, 72 patients (47 from Shenzhen and 25 from Huzhou) met the inclusion criteria. After the 12-week intervention period, 19 patients were excluded due to withdrawal from participation, intake of antibiotics during the trial, COVID-19 infection, and refusal to provide stool samples (Figure S1).

The diagnosis of autism was confirmed by child psychiatrists or rehabilitation physicians, adhering to the criteria outlined in the “Diagnostic and Statistical Manual of Mental Disorders, Fifth Edition, DSM-5”. Inclusion criteria required participants to be aged 3 to 12 years (both males and females) and to meet the diagnostic criteria for ASD. Additionally, all patients were mandated to receive Probio-M8 supplementation and follow balanced dietary guidance throughout the study. Participants also needed to complete the intervention according to the established protocol. Exclusion criteria included: 1) diagnosed malnutrition; 2) severe gastrointestinal diseases requiring immediate treatment; 3) use of immunosuppressants, antibiotics, probiotics, prebiotics, or postbiotics within one month before or during the intervention; 4) severe fever or infection within seven days before enrollment; 5) renal insufficiency and hepatic dysfunction; and 6) known allergies to probiotics or any ingredients used in the study intervention.

In this study, all children diagnosed with autism received a combined intervention of Probio-M8 supplementation and a balanced diet, supervised by a medical doctor. The balanced diet was meticulously designed to provide a targeted caloric intake of 30–40 kcal/kg, calculated based on ideal body weight. For children with obesity or developmental delays, caloric intake was adjusted according to individual needs and the intervention protocol. The diet consisted of 40% carbohydrates, 30% fats, and 30% proteins. Standardizing the dietary structure during the intervention would be crucial for maintaining consistency in dietary intake, particularly given the dietary preferences and unhealthy eating habits often observed in children with autism. This approach helped mitigate any bias due to dietary diversity. We developed an electronic daily diet questionnaire to assess food intake and nutrient consumption across three meals. While recognizing the challenge of capturing accurate dietary habits due to variability and subjective reporting, we mitigated this by closely supervising the participants’ medium-carbohydrate diet. This involved providing detailed meal plans, offering guidance on food choices, and monitoring adherence through dietary logs and bi-weekly calls that recorded consumption frequencies of various food groups, including staple foods, vegetables, fruits, eggs, pulses, dairy products, and meat.

The Probio-M8 used in this study was manufactured by Jinhua Yinhe Biological Technology Co., Ltd. (Zhejiang, China) and provided as a dry powder containing 1.0 × 10^11^ colony-forming units per 2 g. A dedicated individual was responsible for handling, counting, preserving, and distributing the probiotic servings. Subjects and their guardians were instructed to take Probio-M8 once daily with warm water at approximately 40°C half an hour after meals. The intervention lasted for 12 weeks. Probiotics compliance was assessed by counting the remaining doses returned at the end of the study. The combined probiotic and dietary intervention commenced after baseline stool samples were collected. Adverse events, including systemic infection, deleterious metabolic responses, excessive immune stimulation, and gastrointestinal side effects, were recorded throughout the intervention period.

### 2.2. Ethics approval

The study involved eligible families with children diagnosed with autism who provided informed consent via a formal consent form. If the child could make informed decisions, personal consent was also obtained. Only families who provided consent for their children to participate were included in the study. To ensure transparency and accountability, the trial was registered in the Chinese Clinical Trials Registry (http://www.chictr.org.cn) on January 22, 2021, under the registration number ChiCTR2100042512.

### 2.3. Outcome assessments

The primary endpoint of our study was assessed using the CARS (standard version).^24^ The CARS is an observational tool used by clinicians to assess the verbal, behavioral, and perceptual abilities of children with autism. The CARS scale consists of 15 items, including relating to people, imitation, emotional response, body use, object use, adaptation to environmental change, visual response, listening response, taste, smell, touch response and use, fear or nervousness, verbal communication, non-verbal communication, activity level, level and consistency of intellectual response, and general impressions. A score below 30 indicates no autism, while a score between 30 and 36 suggests moderate autism. A score ranging from 37 to 60, with at least five items scoring above three, indicates severe autism.^25^

The secondary endpoint of the study was the GSRS, which utilized a 4-point scale and comprised 15 questions designed to assess symptoms, such as abdominal pain, reflux, indigestion, diarrhea, and constipation. Scores for each domain were calculated based on the average response within that domain.^26^ In this study, we evaluated the CARS and GSRS scores for all children with ASD both before and after treatment, while also documenting any adverse events that occurred during the intervention period. To minimize subjective bias, all questionnaires were administered and completed by medical professionals.

Fecal samples from all participants were self-collected either at the hospital or at home at baseline and three months post-intervention, using sterile stool samplers that contained DNA protection solution (Guangdong Longsee Biomedical Co., Ltd., Guangzhou, China), following the manufacturer’s instructions. Blood samples were collected via venipuncture both before and after the intervention and were analyzed through routine blood tests conducted by the department of clinical nutrition Shenzhen hospital, southern medical university.

### 2.4. Dataset and recruitment of typically developing (TD) control children

To establish a control cohort, we retrieved metagenomic raw sequencing data from 29 TD children in China from the National Center for Biotechnology Information database, under accession number PRJNA688881. Additionally, we recruited 16 TD children from local kindergartens and primary schools through pediatricians, ensuring that both groups were matched for gender and age. Inclusion criteria for the control group were as follows: 1) no diagnosed pathological conditions, including neurological disorders, intellectual disabilities, developmental delays, serious physical illnesses, or gastrointestinal problems; 2) average or near average cognitive, language, social, and motor skill development; 3) no use of antibiotics, probiotics, or prebiotics within one month prior to the study. We recorded information on past medical history, medication history, and basic family details for all participants. To ensure comparability, we carefully matched the TD control children to the participants in this study based on sex, age, and country of origin. This matching process aimed to mitigate the potential impact of these variables on the results and enhance the validity of our findings.

### 2.5. Metagenomic sequencing, binning, and functional prediction

Fecal samples from patients collected at baseline (0 weeks) and after 12 weeks were subjected to shotgun sequencing using the Illumina Novaseq 6000 platform (Illumina Inc., San Diego, CA, USA). Metagenomic DNA was extracted from the stool samples using a fecal DNA kit (DP712; TIANGEN Biotech Co., Ltd., Beijing, China), following the manufacturer’s instructions. A total of 0.97 Tbp of high-quality clean data was generated, averaging 10.69 Gbp per sample (Table S1). All raw metagenomic sequencing samples underwent standardized bioinformatic processing to generate high-quality clean data for subsequent analyses. The generated data was assembled into contigs using Megahit, and contigs larger than 2000 bp were selected for binning using MetaBAT2, VAMB, and DAS Tool. In-house scripts were used to generate metagenome-assembled genomes (MAGs) from the obtained bins.

To assess the quality of these MAGs, CheckM was utilized, classifying them into high, medium, and partial quality based on completeness and contamination levels. High-quality MAGs were further clustered to eliminate redundancy, and the dRep tool (parameters: -pa 0.95 and -sa 0.95) was used to select the most representative genomes from each replicate. This process led to the identification of species-level genome bins (SGBs). The relative abundance of each SGB was calculated using CoverM (https://github.com/wwood/CoverM) with parameters set to: –min-read-percent-identity 0.95 –min-covered-fraction 0.4.

Relevant gut metabolic modules (GMMs) encoded by SGBs were predicted based on information from published literature and the MetaCyc metabolic database. Open reading frames were aligned with the Kyoto Encyclopedia of Genes and Genomes (KEGG) Orthologies Database to annotate the key metabolic modules of each SGB. The distribution of modules involved in synthesis or degradation within the SGBs was identified using Omixer-RPM (parameter: -c 0.66). Furthermore, genes encoding carbohydrate-active enzymes (CAZymes) were detected using dbCAN2.^27^

### 2.6. Phages and bacterial host identification and abundance analysis

VIBRANT is a sophisticated tool designed to efficiently identify a wide spectrum of viruses, including dsDNA, ssDNA, dsRNA, and ssRNA viruses, alongside their bacterial hosts.^28^ This capability allows it to predict prophages that other analytical tools might miss and to clarify the relationships among various viruses in the environment. To investigate the interactions between the phageome and their bacterial hosts, we utilized the functions of VIBRANT for phage identification and host association. First, VIBRANT was employed to detect potential phages in contigs larger than 2,000 bp using default parameters. Subsequently, CheckV was used to evaluate all contigs, and CD-HIT (https://github.com/weizhongli/cdhit) was utilized to identify viral contigs exceeding 5,000 bp. Identification was based on a 95% nucleotide identity threshold, ensuring that at least 80% of the sequences within a cluster met the criteria. The identified viral contigs were further clustered into viral operational taxonomic units (vOTUs). To assess their novelty, these vOTUs were compared against the viral genomes available in the Metagenomic Gut Virus catalog (accessed July 2021). Finally, the relative abundance of vOTUs was calculated using CoverM-contig pipeline (https://github.com/wwood/CoverM) with the following parameters: –min-read-percent-identity 0.95, –min-read-aligned-percent 0.5, –proper-pairs-only, and – exclude-supplementary.

### 2.7. Untargeted fecal metabolomics analysis by liquid chromatography-mass spectrometry (LC-MS)

Freeze-dried fecal samples (0.2 g each) were mixed with 600 µL of a methanol solution containing 2-chlorophenylalanin for 1 min. The samples were then centrifuged at 12,000 rpm for 10 min at 4°C, after which the supernatants were collected and filtered through 0.22 μm membranes for LC-MS analysis. The LC-MS separation was performed using an ACQUITY UPLC BEH amide column (100 × 2.1 mm, 1.7 μm; Waters Corporation, Milford, MA, USA) at a temperature of 25°C and a flow rate of 0.5 mL/min. The mobile phase consisted of a mixture of 25 mm ammonium acetate and 25 mm ammonia in water (A) and acetonitrile (B). The analysis was conducted in both positive and negative ionization modes under electrospray ionization.

The raw data obtained from ultra-high-performance liquid chromatography-quadrupole time-of-flight mass spectrometry were processed using ProteoWizard software and the R package XCMS. Principal component analysis (PCA) and partial least squares-discriminant analysis were applied to identify differential biomarker metabolites between groups. The selection criteria included a variable importance in projection value > 2 and a *P*-value <0.05. To further identify the selected biomarkers and explore potential metabolic pathways, searches were conducted in the Human Metabolome Database (HMDB; http://www.hmdb.ca/), the METLIN Metabolite and Chemical Entity Database (http://metlin.scripps.edu/), the Massbank High-Quality Spectral Database (http://www.massbank.jp), and the KEGG Database (http://www.kegg.com/).

### 2.8. Statistical analyses

Statistical tests and data visualization were achieved using R software (version 4.3.2) and Adobe Illustrator. All data are expressed as mean ± SD. Differences in gender, age, and adverse events between groups were evaluated using the chi-square test. Intragroup differences for primary and secondary outcomes were evaluated using either the unpaired or two-sided paired Wilcoxon rank-sum test. The R packages (e.g., vegan, optparse, mixOmics, ggplot2, and ggpubr) were used to calculate Shannon and Simpson’s diversity indices, and to execute PCA, principal coordinate analysis (PCoA), partial least squares-discriminant analysis, Adonis test, and Procrustes analysis. The Wilcoxon rank-sum test and t-test were used to evaluate statistical differences in other variables between groups, and the Benjamini-Hochberg procedure was used to adjust the *P*-value for multiple comparisons. A *p* < 0.05 was considered statistically significant. Furthermore, correlations among clinical indicators, fecal microbes, and metabolites were identified using Pearson correlation analysis after combining pre- and post-intervention data points.

## 3. Results

### 3.1. Demographic data

**Figure 1.**
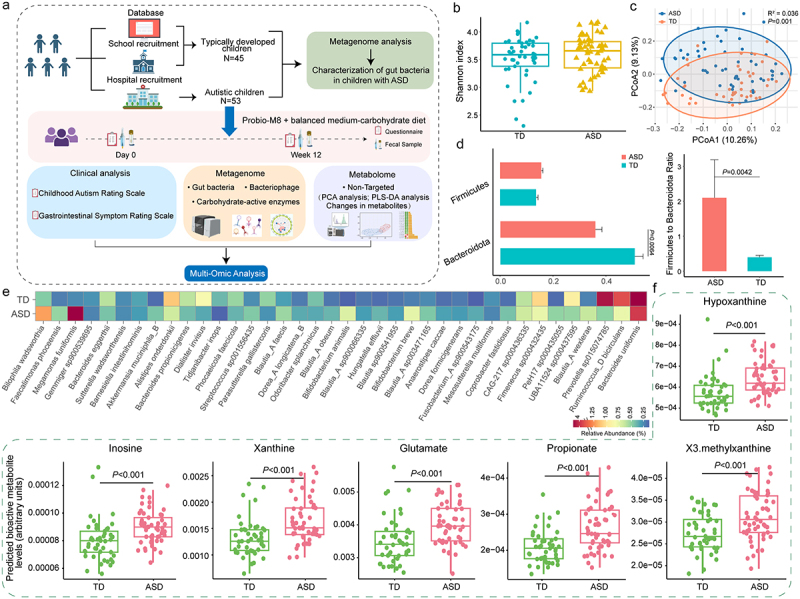
Study design and fecal metagenome analysis of children with autism spectrum disorder (ASD) and typical development (TD).

The study design and demographic characteristics of the participants are shown in Figure 1a and Table S2. The TD group consisted of 45 children, with 29 sourced from publicly available databases and the remaining 16 recruited from kindergartens and primary schools. This group had a female-to-male ratio of 4:41. The ASD group comprised 53 children specifically recruited for this trial, with a female-to-male ratio of 4:49. The mean age of the TD group was 7.68 ± 3.61 years, while the mean age of the ASD group was 6.49 ± 3.08 years. Statistical analysis revealed no significant differences in age and gender between the two groups (*p* > 0.05; Table S2).

(a) Workflow of study design, including the assessment of clinical indicators, fecal metagenomics and metabolomics. (b-d) Comparison of (b) alpha (Shannon diversity index), (c) beta diversity (assessed by principal coordinate analysis using Bray-Curtis distance; Adonis results with 999 permutations), and (d) the relative abundance of Bacteroidota, Firmicutes, and the Firmicutes to Bacteroidota ratio of gut microbiota between the ASD and TD groups. Error bars represent standard deviation. (e) Heatmap showing the significant differential gut species-level genome bins (SGBs) between the ASD and TD groups. The shown SGBs exhibited a *P* < 0.05 (Wilcoxon test) between the two groups following the combined probiotic and dietary intervention. The color scale represents relative abundance, ranging from high abundance (indicated in red) to low abundance (indicated in blue). (f) Levels of predicted differential gut bioactive metabolites responsive to the combined intervention of *Bifidobacterium animalis* subsp. *lactis* Probio-M8 (Probio-M8) and a balanced medium-carbohydrate diet. The results were generated by MelonnPan, a specialized tool for predicting metabolite levels from microbial abundance data extracted from microbial metagenomics dataset. Benjamini-Hochberg procedure-corrected *P* values are shown, with corrected *P* < 0.05 considered statistically significant.

### 3.2. Comparative analysis of the gut metagenomes of ASD and TD children

#### 3.2.1. Altered gut microbiota, predicted gut metabolites and pathways in ASD children

The development of autism is often linked to gut dysbiosis. To explore the relationship between gut microbiota and autism, we comparatively analyzed the fecal metagenomes from 45 TD children and 53 children with ASD.

Evaluation of α diversity using the Shannon index revealed no significant intergroup differences (*p* > 0.05; Figure 1b). However, β diversity analysis using PCoA demonstrated distinct clustering of gut microbiota between the two groups (*R*^*2*^ = 0.036, *p* = 0.001; Figure 1b), suggesting altered structural characteristics of the gut microbiota in children with autism.

We hypothesized that these gut microbiota alterations occurred at various taxonomical levels. Interestingly, our examination of phylum-level microbial distribution revealed a significantly higher Firmicutes/Bacteroidetes ratio in the ASD group (*p* < 0.05; Figure 1d), likely due to a marked decrease in the relative abundance of Bacteroidota. At the species level, we observed reduced abundances of *Prevotella* sp., *Dialister invisus*, and *Bacteroides* sp. in children with ASD, along with increased abundances of *Ruminococcus gnavus*, *Sutterella wadsworthensis*, and *Blautia* sp. (Benjamini-Hochberg corrected *p* < 0.05; Figure 1e; Table S4).

To gain further insight into the alterations in gut metabolic potentials in ASD patients, we utilized the MelonnPan pipeline to predict bioactive metabolites encoded in the microbial metagenomes of both groups, yielding a total of 80 metabolites. Among these, 22 metabolites exhibited significant differences between ASD patients and the TD group. Notably, ASD patients showed significant increases in the predicted abundances of inosine, glutamate, xanthine, and methylxanthine (Benjamini-Hochberg corrected *p* < 0.05; Figure 1f; Table S5).

Moreover, we conducted genome-centric metabolic reconstruction using the MetaCyc and KEGG databases to identify changes in GMMs within the metagenomics dataset, identifying a total of 67 GMMs. Our analysis revealed significant differences in pathways related to amino acid degradation, SCFA metabolism, and vitamin metabolism between the ASD and TD groups. Specifically, pathways associated with arginine degradation, γ-aminobutyric acid (GABA) synthesis, glutamate synthesis, dopamine degradation, and propionate degradation (CQM069, CQM020 and CQM019, CQM006, CQM022, and CQM051,respectively”) were significantly reduced in ASD patients, whereas pathways related to butyrate synthesis, melatonin synthesis, and lysine degradation were significantly enriched (CQM048, CQM003, and CQM056 respectively; *p* < 0.05; Figure S2a), suggesting potential alterations in neurotransmitter levels associated with ASD.

#### 3.2.2. Altered gut phageome in ASD children

**Figure 2.**
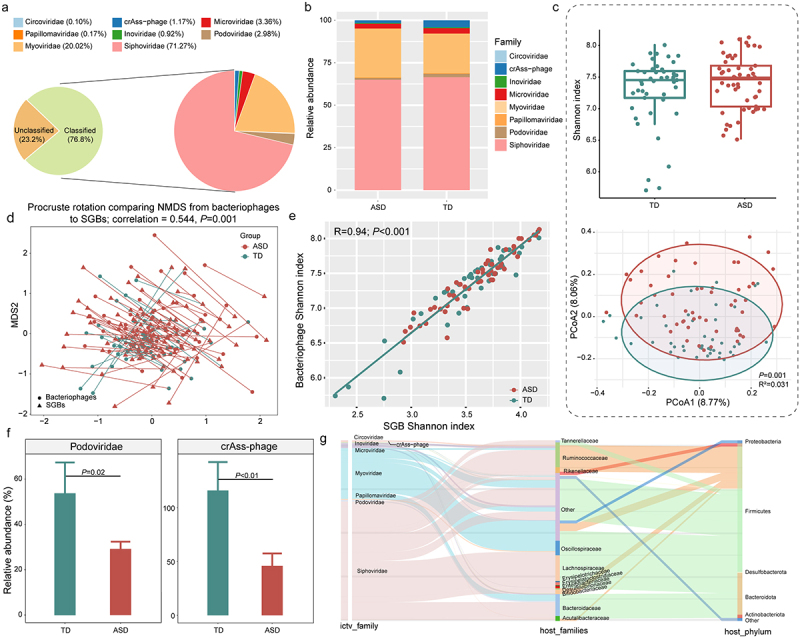
Fecal metagenomes in children with autism spectrum disorder (ASD) and typical development (TD).

The impact of the phageome on bacterial diversity, metabolism, and host health is well recognized, as phages are present in over 80% of bacterial genomes.^29^ We used VIBRANT and CheckV to analyze the gut phageome in TD and ASD children. Our analysis identified a total of 22,418 non-redundant vOTUs, of these 10,259 vOTUs could be annotated against the Metagenomic Gut Virus catalog, with 23.20% classified into 8 known bacteriophage families. The top three families were *Siphoviridae* (71.27%), *Myoviridae* (20.02%), and *Microviridae* (3.36%; Figures 2a,b). While there were no significant differences in the α diversity of the gut phageome between the TD and ASD groups, β diversity analysis revealed distinct inter-group variations (*R*^*2*^ = 0.031, *p* = 0.001; Figure 2c). Notably, Procrustes analysis demonstrated a strong cooperativity between the compositions of the intestinal bacterial and phage microbiota (correlation = 0.54, *p* = 0.001; Figure 2d). Additionally, correlation analysis confirmed a robust relationship between their α diversity (*p* < 0.001, *r* = 0.94; Figure 2e). These results suggest a consistent interaction between the gut bacterial microbiota and the phageome. Interestingly, despite the high similarity in phageome profiles between the two groups, we identified two significantly differentially abundant phage families: *Podoviridae* and *crAss-phage*, which were more prevalent in the TD group (Benjamini-Hochberg corrected *p* < 0.05; Figure 2f, Table S3). These findings highlight important distinctions in phage composition that may be relevant to understanding the microbiome’s role in developmental differences.

(a, b) Family-level taxonomic distribution of the gut phageome for (a) the complete dataset and (b) the ASD and TD groups. (c) Shannon diversity index and principal coordinates analysis (PCoA, Bray-Curtis distance) score plot of the gut phageome for the TD and ASD groups. Results of Adonis tests are shown in the lower right corner of the PCoA plot. (d) Procrustes analysis performed on the gut species-level genome bins (SGBs) and bacteriophages of the TD and ASD groups. (e) Pearson correlation between the Shannon diversity index of the gut bacterial and phage microbiota; (f) The Wilcoxon test was used to evaluate significant differential gut bacteriophages between the two groups. Error bars represent standard deviation. Benjamini-Hochberg procedure-corrected *P* values are shown, with corrected *P* < 0.05 considered statistically significant. (g) Family-level classification of phages and their bacterial hosts at the genus and phylum levels.

To investigate the potential interactions between specific bacterial taxa, gut phages, and active metabolites in TD and ASD groups, we constructed group-based correlation networks (Figure S2b). Our analysis revealed that the interactions among gut microbes in the TD group were more robust and extensive, featuring a total of 309 correlations compared to just 92 in the ASD group. This suggests that TD children possess a more stable gut microbiome and exhibit distinct patterns of microbial interactions compared to their ASD counterparts. Notably, the co-occurrence network in the ASD group consisted entirely of positive correlations. In contrast, the TD group exhibited several negative correlations, including a significant negative correlation between *Phocaeicola faecicola* and the metabolites thymine and glutamate (*p* < 0.05, *r* <- 0.5). These findings underscore important differences in microbial dynamics and their associated metabolites between the two groups, highlighting the complexity of gut microbiome interactions in relation to developmental differences.

Considering that phages infect bacteria, we further explored the relationship between the phageome and their bacterial hosts. Our analysis revealed that *Siphoviridae* was the most abundant phage family associated with bacterial host genomes, predominantly linked to *Ruminococcus, Oscillospiraceae*, and *Lachnospira*. Additionally, *Myoviridae* and *Microviridae* are two widespread and abundant phage families in the human gut, commonly infecting Firmicutes and Bacteroidota hosts, including the taxa *Rikenellaceae*, *Lachnospira*, and *Bacteroides*. Interestingly, *crAss-phage* primarily targeted *Bacteroides* and *Ruminococcaceae* hosts, which aligns with the observed alterations in gut microbiota (Figure 2g).

These findings provide compelling evidence of significant differences in gut microbiota, phage composition, and metabolic pathways between children with ASD and TD children. This further supports the association between gut bacterial dysbiosis and behavioral abnormalities in ASD patients. Moreover, our results underscore the gut microbiome as a potential target for future ASD treatments.

### 3.3. Effects of combined Probio-M8 and medium-carbohydrate diet in ASD patients

#### 3.3.1. Participant characteristics

To investigate the potential role of gut microbes in ASD, we conducted a 12-week intervention involving the probiotic Probio-M8 combined with a balanced medium-carbohydrate diet in a cohort of 72 children aged four to ten years with ASD. During the screening period, eight participants chose to withdraw from the study due to factors such as lack of interest, noncompliance with the diet, or the child’s refusal to cooperate. Throughout the study, four participants were receiving concurrent antibiotic treatment, four were unable to attend visits due to COVID-19, and three declined stool collection. Ultimately, 53 patients successfully completed the 12-week intervention. Among these participants, 30 were from Shenzhen (female to male ratio = 28:2), with an average age of 7.33 ± 3.62 years. The remaining 23 participants were from Huzhou (female to male ratio = 21:2), with an average age of 5.39 ± 1.75 years. At baseline, there were no statistically significant differences in height, weight, or body mass index between the two groups (Table S2).

#### 3.3.2. Improvement in CARS and GSRS scores post-intervention and adverse effects

**Figure 3.**
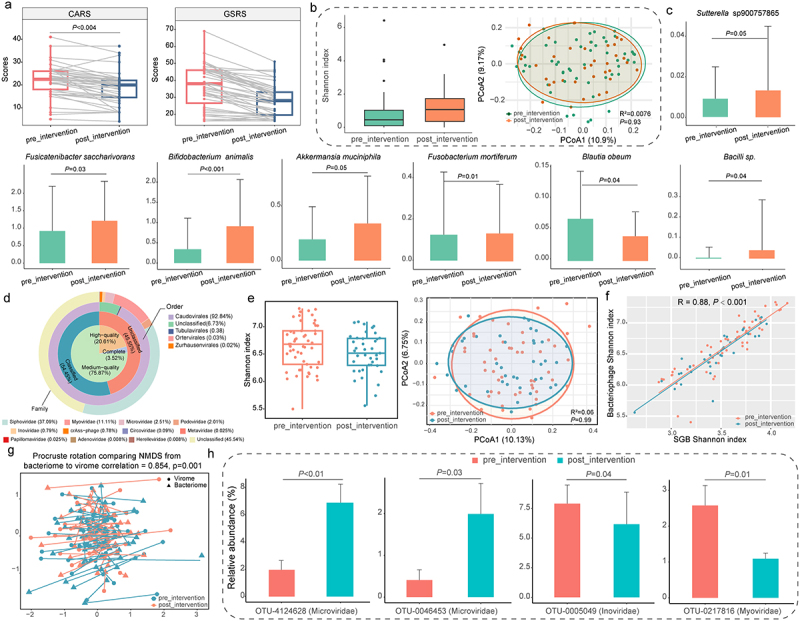
Effects of the combined *Bifidobacterium animalis* subsp. *lactis* Probio-M8 and dietary intervention on improving autism spectrum disorder and gastrointestinal symptoms.

To evaluate the effects of the combined Probio-M8 and dietary intervention on clinical parameters of ASD, physicians assessed participants using the CARS and the GSRS questionnaires (Figure 3a). The CARS scores significantly decreased by 15.22% following the intervention, from 21.82 ± 7.00 to 18.5 ± 7.04 (Benjamini-Hochberg corrected *p* < 0.05). Additionally, GSRS scores improved significantly, showing a 23.0% reduction after the intervention, from 36.43 ± 12.57 to 28.06 ± 9.08 (Benjamini-Hochberg corrected *p* < 0.05; Table S6).

(a) Childhood Autism Rating Scale (CARS) and Gastrointestinal Symptom Rating Scale (GSRS) were used to evaluate the clinical effects. Statistical differences in clinical indicators were assessed using the Wilcoxon test. Benjamini-Hochberg procedure-corrected *P* values are shown, with corrected *P* < 0.05 considered statistically significant. “Pre_intervention” and “post_intervention” represent the baseline (before the intervention) and after the 12-week intervention, respectively. (b) Shannon diversity index and principal coordinates analysis (PCoA; Bray-Curtis distance) score plot with Adonis results (999 permutations) of the fecal bacterial microbiome. Samples from each group are represented by different colors. Results of Adonis tests are shown in the lower right corner of the PCoA plots. (c) Significant differential species-level genome bins (SGBs) identified between the bacterial fecal microbiome before and after the intervention (*P* < 0.05 is considered statistically significant, Wilcoxon test). Error bars represent standard deviation. (d) Circle plot illustrates annotated virus operational classification units (vOTUs), their quality level and completeness (innermost two circles), and classification according to the International Committee on Classification of Viruses (ICTV classification; outermost two circles). (e) Shannon diversity index and PCoA (Bray-Curtis distance; with Adonis results using 999 permutations) of the gut phageome before and after the intervention. (f) Pearson correlation between the Shannon diversity index of the gut bacterial and phage microbiota, revealing a strong positive correlation. (g) Procrustes analysis demonstrating the relationship between SGBs and bacteriophages before and after the intervention, confirming a positive cooperativity between the gut bacterial microbiota and phageome (correlation = 0.854; *P* = 0.001). (h) Significant differential phage families identified between the gut phageome before and after the intervention.

Throughout the study, we closely monitored and recorded any adverse events. Remarkably, only three mild adverse events (diarrhea and bloating) were reported, all of which resolved on their own within 1–2 days of continued intervention. Importantly, there were no instances of nausea, vomiting, abnormalities in routine blood test results, or long-term adverse effects, indicating that the implemented intervention was well-tolerated by patients (Table S2). Furthermore, children with ASD demonstrated high compliance with the intervention.

Overall, these findings provide compelling evidence that the probiotic-assisted medium-carbohydrate diet effectively mitigated symptoms associated with autism in patients, without any serious adverse events.

#### 3.3.3. Changes in gut bacterial and phage microbiota composition post-intervention

The post-interventional changes in fecal microbiota of 53 children with ASD were analyzed using metagenomics analysis. Both α and β diversity analyses (based on the Shannon diversity index, PCoA, and Adonis test; Figure 3b) revealed no significant changes following the intervention, suggesting that the combined probio-M8 and dietary intervention did not result in substantial alterations in the gut bacterial microbiota composition among these children.

We investigated potential species-level changes associated with improvements in clinical indicators by identifying significant differentially abundant SGBs after the 12-week intervention. Specifically, the relative abundances of *Bifidobacterium animalis*, *Fusobacterium mortiferum*, *Akkermansia muciniphila* (*A. muciniphila*), *Fusicatenibacter saccharivorans*, and *Sutterella* sp900757865 increased significantly, while *Blautia obeum* showed a significant decrease post-intervention (Benjamini-Hochberg corrected *p* ≤0.05 for all cases; Figure 3c; Table S7).

In addition to analyzing the bacterial component, we examined the changes in fecal phageome diversity and composition in children with ASD following the intervention. We yielded a total of 20,019 non-redundant vOTUs by comparing our virus dataset with the Metagenomic Gut Virus catalog. Among them 12,089 vOTUs were assigned into 11 bacteriophage families, including 426 (3.52%) complete genomes, 2,491 (20.61%) high-quality genomes, and 9,172 (75.87%) medium-quality genomes. Most of these phage families belonged to the order Caudovirales, with *Siphoviridae* (37.09%), *Myoviridae* (11.11%), and *Microviridae* (2.51%) being the most prevalent families (Figure 3d).

Consistently, α and β diversity analysis showed no significant differences in phage diversity before and after the intervention (*p* > 0.05; Figure 3e). However, interestingly, we observed a significant positive correlation between the Shannon index of the gut microbiota and that of the gut phageome (*R* = 0.88, *p* < 0.001; Figure 3f), which was corroborated by Procrustes analysis (*R* = 0.854, *p* = 0.001; Figure 3g), suggesting a strong correlation between the gut phageome and its bacterial hosts. Furthermore, we examined specific phageome changes associated with the combined probiotic and dietary intervention by comparing the fecal metagenome before and after treatment. Two vOTUs (OTU-4124628 and OTU-0046453) from the *Microviridae* family showed significant increases post-intervention, while OTU-0005049 from the *Inoviridae* family and OTU-0217816 from the *Myoviridae* family exhibited significant decreases compared to baseline (*p* < 0.05; Figure 3h).

#### 3.3.4. Changes in gut microbial metabolic pathways and CAZymes post-intervention

**Figure 4.**
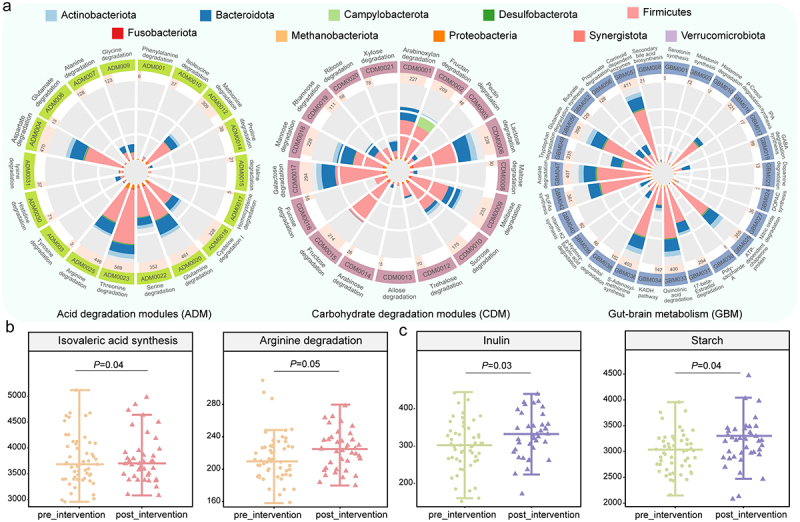
Predicted gut metabolic modules (GMMs) and carbohydrate-active enzymes (CAZymes) of children with autism spectrum disorder before (pre-intervention) and after (post-intervention) the combined *Bifidobacterium animalis* subsp. *lactis* Probio-M8 and dietary intervention.

We further explored the changes in GMMs associated with the intervention using the MetaCyc and KEGG databases. The GMMs were categorized into three groups (Figure 4a; from left to right): amino acid degradation modules (ADM; 18 modules); carbohydrate degradation modules (CDM; 17 modules), and gut-brain metabolism (GBM; 27 modules). Our analysis revealed a total of 62 modules encoded by 419 SGBs across ten phyla, with Firmicutes (68.26%), Bacteroidota (15.99%), and Actinobacteriota (6.68%) being the predominant phyla (Table S8).

(a) Distribution of selected GMMs related to the development, pathophysiology, and immunity of autism spectrum disorder across phyla. From left to right: amino acid degradation (ADM, 18 modules), carbohydrate degradation (CDM; 17 modules), and gut-brain metabolism (GBM, 27 modules). (b, c) The abundances of predicted (b) significantly differential GMMs and (c) substrates of significantly differential CAZyme families that were responsive to the combined probiotic and dietary intervention (*P* < 0.05 is considered statistically significant, Wilcoxon test).

Particularly noteworthy are the GBM modules, which primarily encompass pathways related to polyunsaturated fatty acid synthesis, SCFA synthesis and degradation (including acetate, butyrate, propionate, and isovaleric), tryptophan synthesis, and GABA synthesis. These pathways were encoded by a diverse array of SGBs, predominantly from Firmicutes and Bacteroidota, representing 60.98% and 23.75% of the total SGBs, respectively (Table S4). Although there were no significant differences in the cumulative abundance of the three module groups after the combined probiotic and dietary intervention, we observed increases in the gene abundance of the isovaleric acid synthesis module (CBM034) and arginine degradation module (ADM0025) (*p* = 0.04 and 0.05, respectively; Figure 4b; Table S8).

To gain further insight into the enzyme repertoire responsible for complex polysaccharide metabolism encoded by the fecal microbiota of children with ASD, we annotated genes encoding the CAZyme. A total of 33,959 CAZyme-encoding genes were found across 419 SGBs (Table S9). Most of these genes belonged to the glycoside hydrolase (GH) family (18,806 genes), followed by glycosyltransferase (GT; 8,605 genes), carbohydrate esterase (CE; 3,633 genes), carbohydrate-binding module (CBM; 1,977 genes), polysaccharide lyase (PL; 749 genes), and auxiliary activities (AA; 189 genes). We further predicted the substrates associated with these enzyme families and identified seven common substrates. Interestingly, the cumulative abundance of CAZyme-encoding genes responsible for metabolizing inulin and starch showed a significant increase compared to baseline levels (*p* < 0.05; Figure 4c), suggesting that the imposed intervention may promote the degradation of inulin and starch in the gut of children with ASD.

#### 3.3.5. Changes in fecal metabolome post-intervention

**Figure 5.**
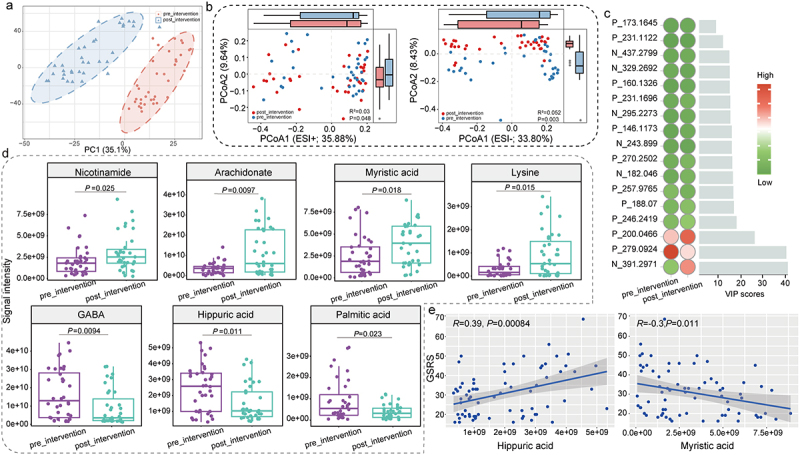
Fecal metabolomes in children with autism spectrum disorder before and after the combined *Bifidobacterium animalis* subsp. *lactis* Probio-M8 and dietary intervention.

We subsequently analyzed the changes in the fecal metabolome of children with ASD following the intervention using PCA. The quality control samples clustered closely on the PCA score plot, indicating strong instrumental stability and data reliability (Figure S2c). The PCA, based on combined metabolomics data from both positive and negative ion modes, revealed distinct clustering patterns associated with the intervention (Figure 5a). Consistent patterns were also observed in the PCoA score plots for both ion modes (*R*^*2*^ = 0.03, *p* < 0.048; *R*^*2*^ = 0.05, *p* = 0.003; Figure 5b). These findings suggest significant alterations in the fecal metabolome structure of children with ASD following the combined probiotic and dietary intervention.

(a) Principal component analysis (PCA) and (b) principal coordinate analysis (PCoA; Bray-Curtis distance; Adonis results with 999 permutations) score plots of the fecal metabolome before (pre_intervention) and after (post_intervention) the intervention; QC represents quality control samples. ESI^+^ and ESI^−^ refer to positive and negative electrospray ion modes, respectively. (c) Heatmap showing changes in significantly different metabolites before and after the intervention. Variable importance in projection (VIP) scores were generated by partial least squares-discriminant analysis to identify metabolites that contributed to the discrimination (cutoff: VIP > 2; *P* < 0.05 was considered statistically significant). The color scale represents metabolite abundance, ranging from high abundance (red) to low abundance (green). (d) Changes in specific fecal differential metabolites responsive to the intervention (Wilcoxon test, *P* < 0.05 was considered statistically significant). GABA means gamma-aminobutyric acid. (e) Pearson correlation scatter plots showing significant correlations between differential fecal metabolites and Gastrointestinal Symptom Rating Scale (GSRS) scores of children with autism spectrum disorder. The gray straight line represents the fitted values, and the shaded area indicates the 95% confidence interval.

Using partial least squares-discriminant analysis, we identified 17 differentially abundant fecal metabolites after the 12-week intervention, applying a cutoff variable importance in projection score > 2 and *p* < 0.05 (Figure 5c). Specifically, there was a significant increase in the levels of nicotinic acid (vitamin B_3_), arachidonate, myristic acid, and lysine, while levels of some other metabolites such as GABA, hippuric acid, and palmitic acid significantly decreased post-intervention (*p* < 0.05; Figure 5d; Table S10). Though not statistically significant, the level of glutamic acid increased postintervention (*p* = 0.055). Furthermore, we explored the relationship between the differential fecal metabolites and ASD-associated clinical symptoms using Pearson correlation analysis. The results revealed a significant positive correlation between hippuric acid and GSRS scores (*R* = 0.39, *p* < 0.01; Figure 5e). Conversely, myristic acid displayed a significant negative correlation with GSRS scores (*R* = −0.3, *p* = 0.01; Figure 5e). These results suggest that specific changes in fecal metabolites following the combined probiotic and dietary intervention are associated with gastrointestinal symptoms in ASD.

Overall, our results demonstrate substantial alterations in the fecal metabolome after the intervention, with specific changes in metabolite profiles potentially linked to the severity of ASD-associated symptoms.

## 4. Discussion

**Figure 6.**
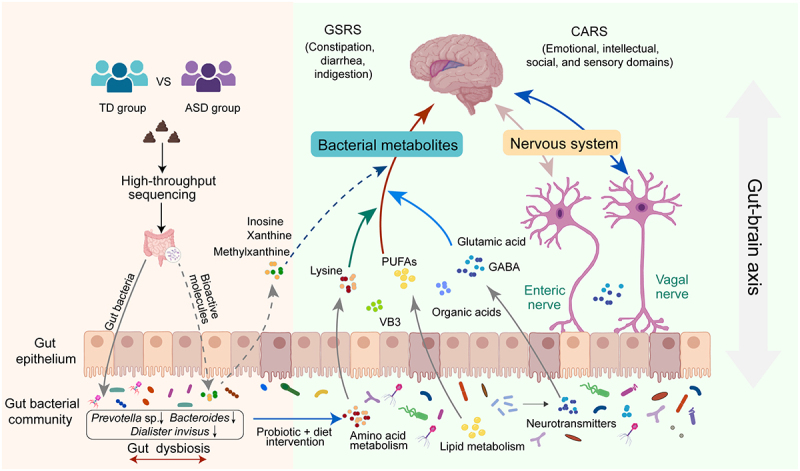
Schematic diagram illustrating the potential probiotic mechanisms of *bifidobacterium animalis* subsp. *lactis* Probio-M8 intervention in autism spectrum disorder (ASD).

Autism spectrum disorder is a complex neurodevelopmental condition that significantly impacts social interaction, communication, and behavior, affecting the quality of life for the afflicted individuals and their families.^30^ Currently, effective prevention and treatment strategies for ASD are limited. However, growing evidence suggests that probiotic treatments targeting gut microbiota hold promise in ameliorating ASD symptoms.^20,31,32^ Nevertheless, previous studies on probiotic interventions have yielded inconsistent results and lacked comprehensive multi-omics data to elucidate the role of probiotics in this context. Additionally, children with ASD often exhibit high food selectivity, leading to deficiencies in essential vitamins and minerals and resulting in unbalanced nutrition. This study aimed to bridge these gaps by examining differences in gut microbiota composition and bioactive metabolites between TD and ASD children. We conducted a single-arm, open-label, pilot study to explore the potential benefits of administering Probio-M8 alongside a balanced diet of medium-carbohydrate in alleviating ASD-associated symptoms. Through integrated metagenomics and metabolomics analyses, we aimed to identify specific biomarkers of gut microbiota and potential bioactive metabolites associated with ASD (Figure 6).

CARS = Childhood Autism Rating Scale, GABA = Gamma-aminobutyric acid, GSRS = Gastrointestinal Symptom Rating Scale, PUFAs = Polyunsaturated fatty acids, TD = Typical development, VB3 = Vitamin B_3_ (nicotinic acid). Dashed lines to represent the predicted.

During the discovery phase of our study, we observed gut dysbiosis in ASD children compared to TD children. Although there were no significant changes in the alpha diversity of gut microbiota in the ASD cohort, PCoA revealed significant alterations in overall gut microbiota structure. Taxonomic analysis indicated a significant increase in the Firmicutes-to-Bacteroidetes ratio in ASD, primarily attributed to a reduction in Bacteroidetes, aligning with previous findings.^33^ On the contrary, some studies have reported a decrease in Firmicutes and an increase in Bacteroidetes in children with ASD.^34,35^ Bacteroidetes are associated with beneficial effects, such as preventing infections from potential gut pathogens,^36^ while an elevated Firmicutes-to-Bacteroidetes ratio has been linked to inflammatory bowel disease and obesity.^37–39^ These results suggest that gut microbiota disruptions may underlie various conditions, including ASD. Additionally, we observed reduced intestinal levels of carbohydrate-degrading genera, such as *Prevotella*, *Ruminococcus*, and *Bacteroides* in ASD patients. These genera are crucial for maintaining mucosal and intestinal epithelial integrity.^40^ Specifically, *Prevotella* and *Ruminococcus bromii* metabolize resistant starch, promote beneficial intestinal bacteria, and facilitate SCFA production.^41^ Similarly, a separate study observed that children with ASD exhibit deficiencies in ileal transcriptional expression for disaccharidases and hexose transporters, indicating impaired carbohydrate digestion and transport, which is accompanied by a reduced Bacteroidetes level and an increased Firmicutes-to-Bacteroidetes ratio.^42^

Bacteriophages play a crucial role in regulating bacterial communities through translocation, induction, and horizontal gene transfer.^43^ A recent study identified ASD as a key factor influencing the composition of the gut-DNA virome.^44^ We also found correlations between specific gut bacteriophages and bacterial taxa in ASD children. For instance, *Siphoviridae* showed a positive correlation with *Prevotella*, while *Faecalibacterium prausnitzii* exhibited positive correlations with *Myoviridae* and *Podoviridae*. Recent research indicates that the intestinal Caudovirales and *Siphoviridae* are positively associated with verbal memory and executive ability.^45^ Notably, the gut bacteriophage composition in individuals with ASD significantly differed from that of TD individuals, particularly regarding levels of *Circoviridae* and *Podoviridae* levels. The parallel changes in the gut phageome and microbiota profiles suggest potential interactions between gut phages and bacterial communities. However, further research is needed to determine how these interactions may influence overall gut microbiota balance in children with ASD.

In addition, a previous metabolomics study has identified abnormalities in purine metabolites, such as uric acid, inosine, and purine products, in children with ASD, providing evidence of metabolic dysregulation in this population.^46^ In our study, we observed significantly increased predicted abundances of inosine, glutamate, xanthine and methylxanthine in individuals with ASD. The interaction between glutamate and glutathione can lead to neuronal dysfunction, and elevated plasma levels of glutathione have been linked to oxidative stress in children with ASD.^47^ Moreover, we identified alterations in specific GMMs in children with ASD, suggesting potential influences of gut microbiota on the central nervous system through various neural, immune, and endocrine pathways. Collectively, our findings provide compelling evidence of gut dysbiosis and metabolic dysregulation in the ASD population.

In the second phase of our study, we examined the efficacy of administering Probio-M8 in conjunction with a balanced diet to alleviate clinical symptoms associated with ASD. By integrating multi-omics data, we identified significant changes in certain gut bacteria, such as *Bifidobacterium animalis*, *Fusobacterium mortiferum*, *A. muciniphila*, as well as phages, including *Microvirida*, *Inovirida*, and *Myoviridae*, following the combined probiotic and dietary intervention. These alterations drove modifications in microbial metabolites, including amino acids, neurotransmitters, lipids, and vitamins, which collectively contributed to mitigating ASD-associated symptoms. To assess the effectiveness of the Probio-M8 and balanced diet combination, we evaluated the severity of autism and gastrointestinal symptoms using the CARS and GSRS scores, respectively. Our results showed a significant reduction in both scores after the combined intervention, aligning with previous research highlighting the potential of probiotics to alleviate ASD symptoms. For instance, one study reported a significant reduction in autism and gastrointestinal symptom severity when probiotics were combined with fructooligosaccharides.^48^ Another study found that administering a mixed probiotic for six months improved certain gastrointestinal symptoms and adaptive functioning in children with ASD, although no significant changes were observed in Autism Diagnostic Observation Schedule scores.^20^ Importantly, we observed no serious adverse events during the intervention period, confirming the safety of this approach for children with ASD and suggesting its potential as a therapeutic strategy for alleviating ASD symptoms.

We then compared the fecal microbial diversity and composition in children with ASD before and after the combined intervention. Interestingly, we did not observe significant changes in gut microbiota diversity, as indicated by the Shannon diversity index. Similarly, PCoA results did not reveal distinct clustering patterns. These findings suggest that the observed symptom relief is not primarily attributed to substantial alterations in gut microbiota diversity, but rather to significant changes in the abundance of SGBs that are relevant to ASD.

We observed a significant increase in the abundance of bacteria with beneficial properties, such as *Bifidobacterium animalis*, *A. muciniphila*, and *Sutterella* sp., following the combined probiotic and dietary intervention. Previous studies have consistently reported decreased levels of *Bifidobacterium* and *A. muciniphila* in patients with ASD compared to the control group.^48,49^ Certain bifidobacterial species have been frequently utilized as probiotics to improve ASD symptoms. For instance, administering bifidobacteria for three months in children aged two to five years with ASD has been shown to enhance autism scale scores, alleviate sleep disturbances, improve speech communication and social networking, reduce hyperactivity, and mitigate gastrointestinal symptoms.^50^

Additionally, *Bifidobacterium longum* has been found to modulate glutamate and GABA levels in the brain and ameliorate microglia activity in the cerebellum, thereby alleviating autistic behavior in rats.^19^ The diminished abundance of *A. muciniphila* in patients with ASD has been linked to increased intestinal permeability.^51^ Furthermore, the negative correlation between *A. muciniphila* and CARS score further supports its potential role in alleviating ASD-related symptom. As a next-generation probiotic, *A. muciniphila* helps maintain intestinal barrier integrity, reduce inflammation, and regulate the central nervous system through its metabolites.^52,53^ *Sutterella* is another important genus of intestinal commensals, playing a pivotal role in regulating mucosal metabolism and maintaining intestinal epithelial integrity.^54^ A previous study has reported a depletion of *Sutterella* in constipated ASD participants, while other reported its indicated altered abundance in the gut of children with ASD, suggesting its potential association with ASD pathogenesis.^55,56^

It is important to highlight the strong correlation between human gut phages and disease occurrence.^57^ The phageome is intricately linked to the bacterial communities it inhabits, with changes in the phageome often lagging behind alterations in these bacterial populations. In our study, we observed a significant increase in the abundance of *Microviridae* following the combined probiotic and dietary intervention. Previous research has reported lower levels of *Microviridae* in patients with gastrointestinal diseases, such as *Clostridioides difficile* infection, Crohn’s disease, and ulcerative colitis, implicating that reduced *Microviridae* levels may be related to disease occurrence.^58,59^ Although the role of the gut phageome is currently understudied compared to intestinal bacterial microbiota, this study, along with recent research, provides interesting insights into the potential role of phages in the gut ecosystem. Understanding these dynamics could pave the way for new therapeutic strategies targeting the gut microbiome and phage interactions in various diseases.

Gut bacteria interact with the brain through neural, immune, and metabolite pathways.^12^ Metabolites and neurotransmitters produced by microorganisms, such as SCFAs, glutamate, and GABA, act as chemical signals that can cross the blood-brain barrier, directly or indirectly affecting the vagus nerve, thereby regulating the central nervous system.^60^ Evidence indicates that disruptions in gut microbial metabolism and metabolite production are common in children with autism.^61^ In this study, we observed higher levels of glutamate and glutamine but lower levels of GABA following the intervention. Glutamate and GABA are excitatory and inhibitory neurotransmitters in the mammalian brain, respectively, and both are directly involved in brain development and synaptogenesis, memory, behavioral regulation, locomotor activity, and gastrointestinal function.^61^ Abnormalities in glutamate receptor genes and dysregulation of glutamate metabolism have been reported in both ASD patients and animal models.^62^ For example, diminished glutamate levels have been associated with the severity of social and behavioral symptoms in ASD.^63^ Reduced abundance of *Limosilactobacillus reuteri* in Shank3 knockout mice was positively correlated with GABA receptor expression in the brain.^64^ Probiotic administration in mice could stimulate a gut microbiota-driven increase in brain glutamate levels.^65^ While GABA itself does not cross the blood-brain barrier, glutamate can be converted to GABA through the enzymatic action of glutamic acid decarboxylase, facilitating neurotransmitter transmission and enhancing gut-brain communication via the vagus nerve.^66^ Numerous studies have consistently highlighted an imbalance in the glutamate and GABAergic systems in relation to ASD.^67^ In line with this, significantly elevated levels of serum GABA, glutamate, and the GABA-to-glutamate ratio have been observed in children with ASD, suggesting potential dysregulation in glutamate and GABA conversion within the brain.^68,69^

Our study also highlights the role of a distinct group of gut microbial neuroactive metabolites, including amino acids, vitamins and polyunsaturated fatty acids, in communication along the gut-brain axis. Following the combined probiotic and balanced diet intervention, we observed elevated levels of lysine, arginine degradation, vitamin B_3_, and arachidonate. These findings are particularly significant considering the established role of B vitamins in regulating oxidative stress and inflammation, as children with ASD have been reported to exhibit lower plasma levels of these vitamins compared to healthy controls.^70^ Notably, a previous study found that vitamin B_3_ can help address mitochondrial dysfunction in ASD.^71^ Vitamin B_3_ contributes to serotonin and dopamine production, as well as the synthesis and conversion of various amino acids and neurotransmitters.^72^ The effects of vitamins on neurological function are likely attributed, at least in part, to their involvement in amino acid metabolism.^73,74^ Vitamin B_3_ supplementation also been shown to stabilize urinary tryptophan concentrations in children with ASD.^75^ Furthermore, earlier research has identified lysine deficiency in the plasma of children with ASD, reinforcing the significance of maintaining amino acid and B vitamin homeostasis for improving ASD symptoms.^76^

Lastly, our findings on altered lipid metabolism in children with ASD align with previous research, characterized by increased levels of acetic acid and palmitic acid, along with decreased arachidonate in plasma.^77,78^ Polyunsaturated fatty acids are essential for infant brain growth and development. Altered fatty acid profiles could be tied to oxidative stress and mitochondrial dysfunction, providing further insights into the metabolic dynamics associated with ASD.^79,80^ Notably, our study revealed a correlation between GABA, hippuric acid, and myristic acid with GSRS scores, suggesting that alterations in these metabolites may represent a potential pathway for alleviating gastrointestinal distress in children with ASD. Hippuric acid is an important product of intestinal microbial metabolism, serving as a marker for the health status and metabolic activity of the gut microbiome.^81^ It is recognized as a uremic toxin linked to advanced chronic kidney disease, contributing to cognitive decline by inhibiting the organic anion transporter 3 at the blood-brain barrier.^82,83^ Moreover, hippuric acid levels have showed positive correlation with microbial diversity and negative association with nonalcoholic fatty liver disease and Crohn’s disease.^84–86^ In individuals with ASD, urinary hippuric acid levels have been found to associate positively with the severity of autism.^87^ However, our study observed a significant decrease in fecal hippuric acid levels following the combined probiotic and dietary intervention. This reduction may reflect changes in ASD-associated symptoms, although the underlying mechanisms warrant further investigation. These findings highlight the complex role of hippuric acid in both health and disease. Additionally, previous metabolomics studies have indicated that young children with ASD exhibit lower plasma levels of myristic acid, a long-chain saturated fatty acid. Notably, higher levels of saturated fatty acids have been positively associated with improved social interactions.^88^ Our results are consistent with this observation, demonstrating increased fecal myristic acid levels after the combined probiotic and dietary intervention. Collectively, these findings suggest that disruptions in gut microbiota may contribute to gastrointestinal and behavioral symptoms in ASD, underscoring the potential of targeted microbial metabolic interventions to alleviate these symptoms.

There are several important limitations to this study that should be acknowledged. Firstly, the relatively small sample size and the limited duration of the study were significant challenges, primarily due to difficulties in recruiting eligible participants with ASD and their families. Consequently, we were unable to include a placebo-controlled group to assess the effects of the intervention without the combined probiotic and dietary intervention. Furthermore, the lack of follow-up post-treatment prevented us from determining whether the observed benefits were temporary or lasting. Despite these limitations, the two-phase study design enabled comparisons of gut microbiota between participants with ASD and a separate age-matched cohort of healthy children. Additionally, the within-subject comparisons conducted before and after the combined intervention provided robust evidence of the efficacy of the imposed intervention. Moving forward, future research should aim to include larger sample sizes and extend the duration of interventions to evaluate the sustainability of the observed benefits, with a randomized controlled design to further validate our findings. Secondly, our assessment focused solely on the overall GSRS score, without analyzing the individual sub-scores; incorporating additional dimensions into the assessment could enhance the comprehensiveness and depth of the study. Further investigation is also necessary to elucidate the specific pathways through which these bacteria exert their beneficial effects. Moreover, the potential role of phages in gut dysbiosis, along with their interactions with bacteria and the host, warrants further exploration.

On a positive note, the integrated approach employed in this study, which combined two clinical measurement scales – namely the CARS and the GSRS – with multi-omics analysis, facilitated an in-depth examination of ASD-associated symptoms. This methodology revealed the intricate interactions between intestinal microbial communities and host physiology. As a result, we identified microbial and bioactive metabolite biomarkers and their correlations with ASD symptoms, offering valuable insights into potential microbial targets and mechanisms for intervention and symptom alleviation. Nevertheless, further research is necessary to deepen our understanding of these microbial targets and their therapeutic potential.

## 5. Conclusions

Taken altogether, our findings demonstrate that the combination of Probio-M8 and a balanced medium-carbohydrate diet effectively reduces clinical scores and alleviates gastrointestinal symptoms in children with ASD. These beneficial effects are likely mediated through the modulation of the gut microbiome and its metabolic pathways involved in the gut-brain axis. This evidence highlights the potential of this combined intervention as a safe and effective strategy for addressing neurodevelopmental disorders. However, further research is essential to decipher the intricate interactions between diet, gut microbiota, probiotics, and ASD, as well as to refine personalized probiotic and dietary interventions for optimal ASD treatment.

## Data sharing statement

Raw sequencing data that support the findings of this study have been deposited in China National GeneBank Sequence Archive of the China National GeneBank DataBase^89^ with accession number CNP0005205 (CNGB; https://db.cngb.org/search/project/CNP0005205/).
